# Communication Styles of Interactive Tools for Self-Improvement

**DOI:** 10.1186/s13612-016-0040-8

**Published:** 2016-06-06

**Authors:** Jasmin Niess, Sarah Diefenbach

**Affiliations:** Department Psychology, Economic and Organisational Psychology, Ludwig-Maximilians-Universität (LMU) Munich, Leopoldstrasse 13, Munich, Bavaria Germany

**Keywords:** Positive computing, Self-improvement technologies, Communication styles, Change success

## Abstract

**Background:**

Interactive products for self-improvement (e.g., online trainings to reduce stress, fitness gadgets) have become increasingly popular among consumers and healthcare providers. In line with the idea of positive computing, these tools aim to support their users on their way to improved well-being and human flourishing. As an interdisciplinary domain, the design of self-improvement technologies requires psychological, technological, and design expertise. One needs to know how to support people in behavior change, and one needs to find ways to do this through technology design. However, as recent reviews show, the interlocking relationship between these disciplines is still improvable. Many existing technologies for self-improvement neglect psychological theory on behavior change, especially motivational factors are not sufficiently considered. To counteract this, we suggest a focus on the dialog and emerging communication between product and user, considering the self-improvement tool as an interactive coach and advisor.

**Methods:**

The present qualitative interview study (N = 18) explored the user experience of self-improvement technologies. A special focus was on the perceived dialog between tool and user, which we analyzed in terms of models from communication psychology.

**Results:**

Our findings show that users are sensible to the way the product “speaks to them” and consider this as essential for their experience and successful change. Analysis revealed different communication styles of self-improvement tools (e.g., helpful-cooperative, rational-distanced, critical-aggressive), each linked to specific emotional consequences.

**Conclusions:**

These findings form one starting point for a more psychologically founded design of self-improvement technology. On a more general level, our approach aims to contribute to a better integration of psychological and technological knowledge, and in consequence, supporting users on their way to enhanced well-being.

## Background

Technologies for self-improvement have become an increasingly popular consumer product and tool of healthcare providers (e.g., Diefenbach et al. 2016). In line with the general scope of positive computing and technology for well-being and human potential (e.g., Calvo and Peters 2013; Sander 2011), such products want to support their users in reaching personal goals like living more healthy, doing more sports, or taking the appropriate time for reflection and thankfulness during the day. SIMA, for example, is a mobile app which supports the user in integrating mindfulness into everyday life. Other forms of self-improvement tools are internet-based interventions in the context of occupational healthcare. For example, an online training for teachers enabled a significant reduction of sleep problems as well as an increase in recreational behavior and mental detachment from work (Thiart et al. 2015). Such examples reflect the high potential of technology to support human well-being and make it appear as a promising complement to more traditional forms of physical and mental healthcare provision (Monkaresi et al. 2013; Wiencke et al. 2014).

Apart from these promises, the product category of self-improvement tools also comprises some tricky challenges, especially when it comes to questions about concrete interaction design. This could be, for example, the manner of feedback which should be given to the user, the visualization of progress (or relapse) to create long term motivation, or the proper timing of feedback (see also IJsselsteijn et al. 2006). As Kanis and Brinkman (2008) phrased it: “There is clearly an opportunity to employ technology for positive change, but how this can be achieved is more difficult to determine”. As focusing on the complex and sensible issue of human behavior, the design of self-improvement technologies requires an interdisciplinary perspective and knowledge from different fields (e.g., psychology, design, human–computer interaction) must be integrated for the best solution possible (Calvo et al. 2014). In order to support positive change, not only technical solutions are needed to initiate positive behavior (e.g., reminders, feedback), but also a psychologically founded and motivating conceptualization of communication from product to user, and an adequate representation through design.

However, current reviews show that this is often not the case. For example, a review on physical activity apps showed a limited number of utilized behavior change techniques and a relative disregard of motivational compared to educational factors (Conroy et al. 2014). Self-improvement technologies such as *Sleepcare* (Beun 2013; Beun et al. 2016), explicitly built on theoretical knowledge from psychology and coaching, are rather exceptions. *Sleepcare*, for example, negotiates with users about an adequate amount of sleeping hours in parallel to a coaching process, copying the phases of *alignment*, *plan and commit*, and *task execution*. The moment a person chooses to improve their self and enhance their well-being with the help of an interactive product, the self-improvement tool transforms to an interactive coach and advisor with a responsible role. As one participant in the study by Beun et al. (2016) put it: “It sounds kind of funny, but I had the feeling of a ‘bond’ with my coach, although I am very much aware that it’s just an algorithm”.

We believe that an explicit understanding of the interaction between tool and user as an “act of communication” appears of vital importance for a sensible design of self-improvement technologies. Considering that, the interaction between tool and user actually represents a form of “therapeutic intervention”, i.e., a purposeful and systematic support of positive change, the product-user-relationship seems to be an important basis for this. Like in traditional face-to-face settings for coaching and therapy, where the emerging dialog between coach and client is acknowledged as an essential factor for the success of change (e.g., Lutz 2010), this idea may be transferred to the dialog between self-improvement technologies and their users as well.

In the present paper, we explore the relevance and potential of communication between self-improvement tools and their users with the help of models from communication psychology. A first question is whether different self-improvement technologies use different styles of communication, and whether users perceive and discuss these. Furthermore, we elaborate on the emotional consequences of different styles of communication on the user’s side and possible relations to specific purposes or needs. The following section summarizes relevant background theory on the relevance of dialog in human–computer interaction (HCI) as well as therapeutic settings and presents relevant models from communication psychology. Afterwards we present an interview study with 18 users of self-improvement tools, reporting on their experience and the perceived communication of the used technology. Finally, we discuss the present study’s limitations and important issues for future research.

## Theoretical Background

### The Relevance of Dialog and Interaction—in HCI and Therapy

When interacting with technology, people often show reactions that correspond to behavior towards living beings (Fogg 2003; Nass et al. 1994). In other words, people interact with technology in a social way (Nass et al. 1994). For example, human–robot-interaction can trigger similar emotional and psychological reactions as human–human-interaction (Jung et al. 2015). People are able to differentiate different communication styles used by robots, whereby user preferences for communication styles depend on the user’s cultural background (Rau et al. 2009). For example, Chinese participants rated robots as more likeable and trustworthy when they used an implicit communication style—a finding that might also apply to human–human-communication (Rau et al. 2009). In an experiment of Nass et al. (1994) participants reacted to different computer voices as though they were different social actors, regardless if they were on the same or different computers.

The form of communication thus appears as a central aspect for the overall experience in human–computer interaction. Note, however, that verbal dialog is only one aspect that may affect the perceived communication or character of a product. For example, also written language (displayed on a computer screen) can evoke a more dominant or submissive impression of a computer’s personality (Nass et al. 1995). And also different forms of physical interaction and related interaction attributes (e.g., slow vs. fast, gentle vs. powerful) come with particular experiential qualities (see also Lenz et al. 2013), and may result in attributions of character such as attributions of interaction character like stubborn (Djajadiningrat et al. 2007), more dominant or more elegant (Desmet et al. 2008).

In sum, there are many parallels in the perception of technology “speaking” to people and human communication, which suggests the form of dialog as a relevant design factor. This especially pertains to the sensible field of technologies for self-improvement where interacting with a product becomes a form of digital therapy. A good relationship and fruitful communication between tool and user seems to be vital for change success.

In classical therapy, dialog has always played an important role. Sigmund Freud for example was using the dialog between himself and his patients as a central tool for his psychoanalysis (Friedman 1987). Jung also admitted that the quality of the dialog is important, if the psychotherapy should be effective and equated therapy with a dialog between two persons (Friedman 1987). Also Harlene Anderson, a US-American psychotherapist and founder of postmodern psychotherapy, emphasizes the dialog between therapist and patient as essential enabler for personal growth and well-being (Anderson 1999). Also approaches to self-improvement and development opportunities in other areas, apart from therapy, e.g., leadership, cannot be imagined without a good and functioning dialog (Regnet 2003). All in all, not only content but also the style of communication is crucial for the resulting perceptions and actions of the communication partners.

### Psychological Styles of Communication

In communication psychology, different models and taxonomies have been suggested to describe and distinguish different communication styles. In the following, we focus on two models, which later built the basis for our categorization scheme of communication styles of self-improvement technologies.

Schulz von Thun (1989) postulates eight different styles of communication: the *needy*-*dependent* communication style is about getting help and support from other people. People with this communication style present themselves as weak and helpless. The *helpful* communication style can be described as helpful, strong and resilient. Individuals with the *selfless* communication style consider themselves as irrelevant. Their feelings of being useful only manifest through the work they do on behalf of others. The *aggressive*-*demeaning* style of communication concentrates on the mistakes and weaknesses of others. For individuals with the *self*-*praising* style of communication it is important how they appear to other people. They constantly try to present themselves in the best possible light. People with the *determining*-*controlling* style of communication attach importance to rules, and they aim to control other human beings, as well as their environment. The *self*-*distancing* communication style is, as the name suggests, characterized through the importance of distance and a preference for a rational perspective. Individuals with the *communicative*-*dramatizing* style of communication like to be in the center of attention and tend to dramatize in their elaborations. However, the eight styles do not form distinct categories in those an individual uses only one style of communication all the time. Instead, Schulz von Thun (1989) suggests that most people combine tendencies of different styles.

Hofmann (2011) based his taxonomy on the communication model of Schulz von Thun (1989). He narrowed the taxonomy slightly down to facilitate a better differentiation between the different styles, ending up with seven different styles of communication: Individuals with the *self*-*centered* style of communication are energetic and like to be in the center of attention. People with the *dramatizing* communication style live in a world full of color and intensity. They often show spontaneous and impulsive behavior. The *cooperative* style of communication is about being helpful and caring. The *diligent* communication style emphasizes the importance of principles. People with this style of communication aim to do everything “the right way”. Individuals with the *critical* style of communication display a gap between apparent and hidden behaviour patterns. At first glance they behave similarly to people with the *cooperative* communication style. But at a second glance they tend to behave rather sceptical and passive aggressive. Individuals with the *rational*-*distanced* communication style do not want to get to close to their fellow human-beings. It is important for them to stay objective and in control of their emotions. The *sensitive*-*avoiding* communication style can be characterized as kind, cautious and controlled.

### Communication Styles in the Context of Self-Improvement Technologies

For several reasons, we think that the well-established taxonomy of communication styles by Schulz von Thun (1989) and the later simplification by Hofmann (2011) might also be a useful lens on the communication of technologies for self-improvement. The definitions of styles are precise but still broad enough to integrate various aspects and ways a product might “speak” to the user (e.g., voice output, textual reminders). Furthermore, Schulz von Thun (1989) puts emphasis on the situational context and the personality of the communicating person. We think that these two aspects are also essential aspects within a process of change or self-improvement.

Based on an expert discussion among psychologists on parallels and central characteristics of the different communication styles, we consolidated the styles, suggested by Schulz von Thun (1989) and Hofmann (2011), into six communication styles. This was to provide a compact and convenient taxonomy for analysis, without neglecting central aspects of communication. Table 1 presents the six summarized communication styles and short descriptions, which we used to analyze user reports in the domain of self-improvement technologies (see next sections).

**Table 1:** Summarized styles of communication and short descriptions

Style of communication	Short description
Helpful-cooperative	Is about helping and caring for other people
Diligent-determining-controlling	Is about guiding and controlling the environment
Rational-distanced	Considers all aspects from an objective, austere perspective
Critical-aggressive-demeaning	Focuses on imperfections and weaknesses of other people
Self-praising-dramatizing	Is impulsive and loves being the center of attention
Selfless-sensitive-avoiding	Is always kind and controlled

## Research Questions

Our analysis of the communication between self-improvement tools and users focused on three main research questions. (1) At first, we were interested to see to what degree users’ actually perceive the interaction with technology as a form of communication, and whether we will be able to detect different communication styles in the user reports on their experiences with self-improvement technologies. (2) A second research interest was on the emotional consequences of different styles of communication for the user. This is based on the vital role of emotional change in classical therapy (Gerrig et al. 2011) and theoretical models of change processes (e.g., intentional change theory, Boyatzis 2006). (3) Finally, we aimed to explore possible relations between different communication styles and individual purposes or needs, e.g., whether the preferred style of communication depends on the kind of goal a user wants to achieve. In sum, we aimed to explore to what degree communication style may affect and influence the effectiveness of self-improvement tools, and whether models from communication psychology are useful to describe and (taking a future perspective) inspire the design of such technology.

## Methods

### Participants and Procedure

We conducted 18 semi-structured in-depth interviews with users (nine female, nine male) of self-improvement-technologies (e.g., mobile apps). The mean age of the participants was 29.83 years (SD 10.48, min = 16, max = 58). The sample included students in different fields (e.g., economics), employees in various areas (e.g., public sector) and one school student. Each participant was given a pseudonym and was assured of anonymity and confidentiality. Study participation was voluntarily and there was no incentive. The interviews were conducted in a quiet environment and special emphasis was placed on a relaxed and open atmosphere. All interviews were held by the first author, holding a master’s degree in psychology and being trained in interviewing techniques.

Each interview started with some broad, open questions about the theme of interest. The participants were asked to name some interactive tools for self-improvement they know or recently had tried. In succession they were asked to pick one they used themselves and to describe it in more detail. Thereupon the interview focused on the perception of character and communication-style of this tool. In the last part of the interview, the questions got more specific and concentrated on different styles of communication, orientated on the taxonomy listed in Table 1. Note that this structure helped to manoeuvre through the interview, but had not necessarily to be followed. To gain a deeper understanding of the theme it was the responsibility of the interviewer to enable the narrative thread of each participant and to guide him back to the subject of interest (Witzel 2000). The interviews were audiotaped and lasted between 20 and 60 min (mean duration = 37.3 min, SD 13.15).

### Analytic Strategy

The interviews were fully transcribed with the transcription software f4. Data analysis included two steps. The first step was a non-focused, summarizing content analysis (Mayring 2014) across the whole of interview data, including the following sub-steps:

- Paraphrasing of content-bearing text passages to the intended abstraction level (note that we checked every time before we wrote a new paraphrase down, if it is possible to include a new paraphrase to those who have been made already.
- Collation of the new paraphrases as a category system and the re-testing of the paraphrases as a category system.

The non-focused, summarizing content analysis (Mayring 2014) was conducted to detect potential further recurring issues of relevance related to the topic of using self-improvement tools. Categories were communication between the tool and the user, usage motivation, frequency of use, the goal of the user and the consequences for the user and areas of use.

In a second step we implemented a structuring content analysis (Mayring 2014), with a focus on the product-user-dialog. We used the six styles of communication (Table 1) to categorize those interview statements referring to the communication/dialog between product and user. Short descriptions and keywords related to the six styles of communication facilitated the coding process. Besides the six styles, the category system also included an open category in case there would be content which is unmatchable to the categories of the theory-based taxonomy. However, results showed that all statements concerning the communication between the interactive tool and the user could be assigned to one of the six theory-based categories.

The reliability of the allocation of statements to the categories of the category-system was verified by passing the allocation task to an independent rater holding a PhD in physics and a bachelor’s degree in philosophy. The independent rater again processed 50 % of the material, i.e. nine interviews, the interrater agreement was satisfying (Cohen’s Kappa = .85).

## Results

**Fig. 1:**
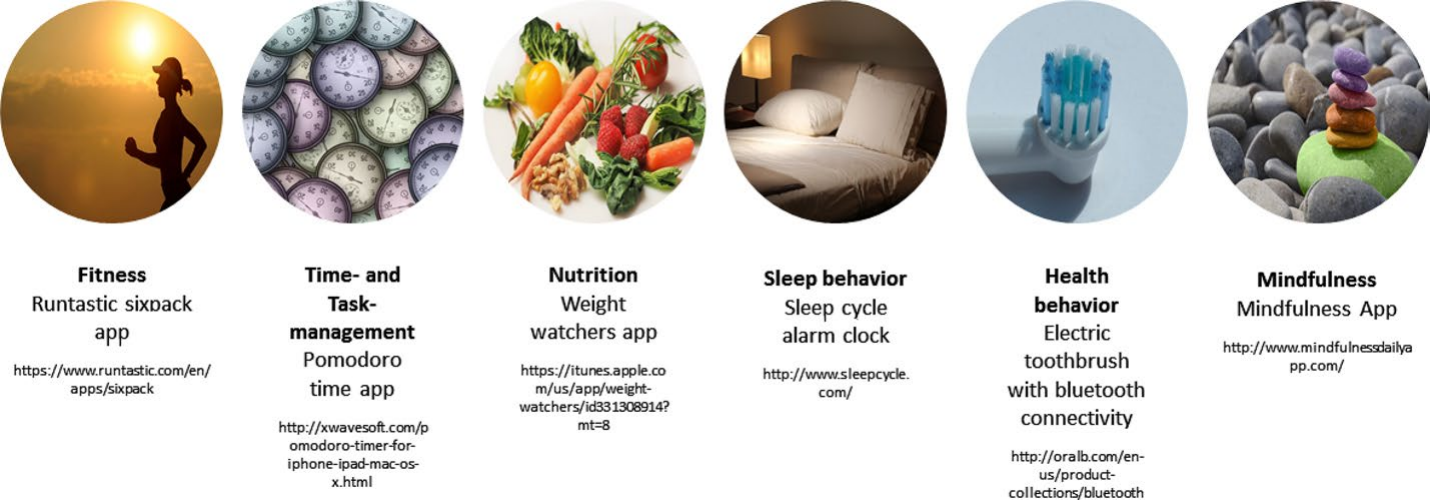
Self-improvement categories and sample products

All statements regarding the communication between the interactive tool and the user were successfully assigned to the six different styles of communication (see Table 2 for sample statements). General statements on the importance of dialog further underlined the relevance of the topic. Thus, regarding research question 1, it can be concluded that users perceive the interaction with self-improvement technology as a form of dialog about their goals and progress and are sensible to the style of communication. Also, the interviews revealed particular emotional consequences for each of the styles (research question 2) and relations to individual goal characteristics, such as long term versus short term goals (research question 3). The participants mentioned self-improvement technologies from various fields. All six fields of usage and one mentioned sample product for each category are presented in Fig. 1.

In the following, we describe such findings in more detail. We open with the general relevance of dialog. After this, we discuss the prevalence of the six different styles of communication and central aspects. Emotional consequences and relations to goal characteristics are exemplified by two styles of communication, i.e., helpful-cooperative and critical-aggressive-demeaning.

### General Relevance of Dialog

Interview data confirmed the dialog as a central element within the interaction between self-improvement product and user. Participants noticed the way a product speaks to them and perceived it as an important factor for long term use and their process of self-improvement. Julian for example stated that the presetting somehow determines how the app talks to you.

*“What matters is, what kind of presetting you are selecting. You are able to choose how you want to be addressed or contacted by the app.” (Julian)*

In line with our conceptualization of self-improvement tools as an interactive coach and a partner on the way to improved well-being, Simone explained:

*“Some people want to use the tool because they don’t have a training partner. So the app is becoming their training partner or their nutrition coach. It’s different when the app counts your working hours or your calories. It’s the app as some kind of calculator or machine, versus the app as some kind of partner.” (Simone)*

Also, participants saw a relation between the form of dialog and a successful usage history. For example, Dinah reflected:

*“I usually quit the apps after some time, because something is bothering me, I don’t know, maybe it has actually something to do with the communication.” (Dinah)*

## Different Styles of Communication

The most prevalent styles were the helpful–cooperative, the diligent-determining-controlling and the rational-distanced style of communication. The helpful-cooperative style of communication was mentioned by 83 % of the participants. The least represented communication style was the selfless-sensitive-avoiding, mentioned by only two out of 18 participants (11 %). Participants’ narrations revealed typical reoccurring issues and characterizations for the different styles, such as “a personal trainer” for the diligent-determining-controlling communication style or “a neutral assistant” for the rational distanced style of communication. Table 2 presents the six summarized communication styles, the frequency of total mentions and number of participants mentioning each style, as well as exemplary statements.

**Table 2:** Six styles of communication in self-improvement tools

Style of communication	Frequency:# mentions (# participants)	Central aspects, characterizations	Sample statement
Helpful-cooperative	41 (15)	Friendly instructor, motivator, caring	“Then it said something like: “It’s not that bad. It happens. Have you checked your water intake and your vegetable consumption? Keep that in mind. If this doesn’t help, try eating less fruit and more vegetables.” Advice like that”
Diligent-determining-controlling	33 (13)	Personal trainer, strict	“I wouldn’t call it a drill sergeant, but it is a bit like a personal trainer, who tells you to work out now, about the upcoming exercise and how long it will take and when to take breaks, but it’s also motivating”
Rational-distanced	21 (11)	Neutral assistant, reasonable	“The app is quite neutral and rational, less personal”
Critical-aggressive-demeaning	17 (8)	Drill sergeant, mean, tough	“It just shows you your slowest time of your entire run and there is a tortoise symbol and the fastest part has […] a hare symbol or something similar. That is somehow mean and rubs salt into the wound”
Self-praising-dramatizing	11 (7)	Drama queen, exaggerating	“Today you have exceeded your daily limit. And then there is always this notification: “If you ate that or that over the next few days and weeks, you would weigh so or so much.” So it likes to dramatize things as well”
Selfless-sensitive-avoiding	2 (2)	Cautious, timid	“It (the product) tries to address this uncomfortable topic in a very kind, reserved way”

### Emotional Consequences and Relations to Goal Characteristics

Regarding emotional consequences (research question 2) and relations to goal characteristics (research question 3), clusters of communication styles with similar consequences could be detected: For example, the diligent-determining-controlling, the rational-distanced and especially the helpful-cooperative style evoked mainly positive emotions. On the contrary, the consequences of the critical-aggressive-demeaning style of communication were described negative by some people and positive by others.

Regarding relations to goal characteristics, the data suggests that the preferred style of communication is linked not so much to the field of usage as to the individual goal of the user. For example, it seems more relevant in what time you aim to achieve a goal (e.g., 2 weeks versus 6 months) than in what area you want to improve (e.g., sports, nutrition). More specifically, participants’ statements suggest that long-term goals go well with “softer” styles of communication (e.g., helpful-cooperative. rational-distanced) whereas short-term goals with the need for quick results go well with “harder” styles of communication (e.g., critical-aggressive-demeaning).

In the following, these relations are exemplified in more detail for two styles of communication. As useful representations of the broad spectrum of communication a tool can provide and in order to highlight the different consequences; we chose to contrast the helpful-cooperative style and the critical-aggressive-demeaning style.

### Helpful-Cooperative Style of Communication

The helpful-cooperative style of communication is about helping and caring for other people. The communication style is polite, diplomatic, as well as resilient. Dinah, for example, described how she got some advice from her interactive product for self-improvement, in a nice and polite way:

“…for example when it [the weight of the user] had stagnated or increased in some kind of way. Then it said something like: “It’s not that bad. It happens. Have you checked your water intake and your vegetable consumption? Keep that in mind. If this doesn’t help, try eating less fruit and more vegetables. ”Advices like that.” (Dinah)

Typically, the app is described as a friendly and motivated instructor, who also provides helpful feedback, for example, at the end of a run.

“…like a fitness instructor […]. Somebody that keeps hopping on one foot and then the other and wants to take me out for a run and runs beside me and keeps saying: “You’re doing fine. Keep up the good work!” When using the app it’s also like that: now that I use an instructor in the app, a real instructor gives you feedback in the end and says: “Hey, good job seeing it through. That was a really good run.” That’s probably why I imagine it that way.” (Flora)

Eight participants explicitly referred to emotional consequences of that helpful-cooperative style of communication. The mentioned emotions were mainly positive, as demonstrated by the following statement.

“It [the helpful-cooperative style of communication] has a positive effect. The emotions are… well I feel motivated and encouraged and afterwards I’m very pleased with my performance.” (Flora)

At this point it is important to emphasize that all the eight participants who had experienced the helpful-cooperative communication style as positive pursued long-term goals (e.g., pursuing a healthier lifestyle, managing and organizing free time, working hours and time for studying over a couple of semesters in order to achieve the best possible outcome).

### Critical-Aggressive-Demeaning Style of Communication

The critical-aggressive-demeaning style of communication focuses on imperfections and weaknesses of other people. People with this style of communication use the deficiencies of other people to make them feel small and insignificant. Within the domain of self-improvement tools, this could be, for example, a critical side blow from a running app:

“Sometimes it can be a bit mean, there is one track where you have been your slowest and it will show you a tortoise symbol and stuff like that […]. It just shows you your slowest time of your entire run and there is a tortoise symbol and the fastest part has a […] hare symbol or something similar. That is somehow mean and rubs salt into the wound.” (Laura)

Similarly, Fabian talked about the demeaning style of communication of the abdominal muscle training program:

“There is this abdominal muscle training program, also by Runtastic. That one is definitely male. That’s what I’d attribute to it […]. Also this program shows no mercy, […] you are lying there and your legs are above your head and you think: “Actually, this hurts a lot” It just keeps going on and on and on” (Fabian)

Four participants reflected on the emotional consequences of the critical-aggressive-demeaning style of communication, showing ambivalent perceptions. The ambivalence towards this communication style is reflected in the following statement.

“Exactly, I am not that kind of/for example Chris is accessible for this, he is the bootcamp kind of guy, being yelled at and so on. He becomes totally aggressive, but very motivated at the same time. And I am becoming defiant and lose interest completely.” (Dinah)

The goals of the users mentioning this communication style were mixed. Five out of eight users pursued long-term goals and attributed negative emotional consequences to the critical-aggressive-demeaning communication style. Three of the eight users pursued short-term goals and described the emotional consequences as “not pleasant” but “very effective”, so it seems to be the right form of communication for some participants in order to reach their goals.

## Discussion

The findings of the present interview study (N = 18) revealed, that users perceive the interaction with self-improvement tools as form of communication and they are able to distinguish between different communication styles (research question 1). Furthermore, the way a self-improvement product speaks to its user can possibly influence the effectiveness of the self-improvement tool. There seems to be a connection between the specific style of communication and emotional consequences of the user (research question 2). Additionally, the individual goal a user is pursuing seems to be connected to the preference of one communication style over another (research question 3). In order to analyze our data we used typologies of communication psychology. We see our findings as one possible starting point to inform designers of self-improvement technologies about the importance of communication aspects. Beyond the general relevance of the perceived communication, a particular important aspect to consider seems to be the type of self-improvement goal and its particular characteristics.

Our findings suggest a connection between the type of goal users are pursuing (long-term goals vs. short-term goals) and the preferred style of communication (e.g., helpful–cooperative vs. critical-aggressive-demeaning). On a more general level, it would be interesting to compare communication styles making use of intrinsic motivation techniques and communication styles making use of extrinsic motivation techniques (Monkaresi et al. 2013). While extrinsic motivation techniques may work best for fast changes (i.e., short-term goals), long-term goals may require communication techniques with a focus on intrinsic motivation. For example, in the present study, one participant had the short-term goal to get a six-pack as fast as possible, to look good on the beach vacation. He deliberately chose a very “strict” mobile app with a hard communication style (i.e., critical-aggressive-demeaning) in order to achieve this goal in the quickest possible way. Several users pursuing short-term goals described the critical-aggressive-demeaning communication style as unpleasant but effective. In contrast, long-term goals might require another form of communication. For example, one participant pursued the long-term goal of playing the guitar on a regular basis. She was intrinsically motivated and preferred the rational-distanced communication style, because she does “not need a motivator”, she only needs “a little support organizing her practice time”. One possible implication could be to offer alternative versions of the same tool, providing the “right” style of communication, depending on the user’s specific goal.

Apart from the connection between the kind of goal a user pursues and the preference of one communication style over another, personality traits may also affect such preferences. Maybe there are some users that would consider the critical-aggressive-demeaning style as quite effective, but are not able to handle that kind of interaction.

However, a tricky question seems to determine which communication style is the “right” one for which user. One position could be to assign this responsibility to the user, which was also what some of our participants demanded. Anna, for example, wished for a product that *“You can personally set how you like it, to personalize your product. I mean, I think it would be good if you could really set the tool exactly how you like it, to adapt it to your needs”.* This is in line with the assumptions from solution-focused coaching (Bamberger 2015), seeing the client as the expert for his life and knowing best what he or she needs to flourish. Indeed, there are also some examples of such self-improvement tools, putting a high responsibility in goal setting and self-regulation to the user. For example, the mobile app MoveMyDay allows the user to set their own, realistic goals concerning their physical activity, in order to lead to a higher performance (Herrmanny et al. 2016). However, there are also studies suggesting that users/coaches might not be the best experts for themselves when it is about choosing an effective approach for change. For example, studies in the field of coaching showed no advantages for coaching success depending on whether the intervention/way of goal attainment was self-selected or not (Silberman 2007).

A possible midway between one-fits-all solutions and total customization of self-improvement tools could include a brief analysis before the intervention starts, asking the user a few questions about their motives and their goals (Burke and Linley 2007). Self-improvement technologies thus could supply general basic approaches and complement those with options for individualization for single aspects (Desmet and Pohlmeyer 2013). The individual adaptation of the “coaching approach” can make the user feel more included and respected, and in turn increase the general commitment towards the intervention (Bamberger 2015).

## Limitations and Future Research

The study is subject to some limitations, which need to be addressed in future research. First, the present categorization of six styles of communication, based on models from communication psychology, and here applied in the domain of self-improvement tools, must be seen as preliminary. Though it served as a helpful frame to categorize participants ‘descriptions of their products’ perceived communication with satisfactory interrater agreement, next steps of research should critically test and further develop the present taxonomy. For example, to make it a more convenient taxonomy, further studies could aim for a reduction to the most common communication styles in the area of interactive technology (or more specifically, self-improvement tools). Also, we would aim for clearer descriptions and definitions of the different communication styles in the technology domain, to make it easy to apply for other researchers as well.

Another limitation of the present study refers to the sample, mainly built of students younger than 35 years. Though self-improvement tools are indeed especially popular among younger users (Rau et al. 2012), further studies among more heterogeneous samples are recommendable. Especially the group of the elderly could profit from self-improvement technologies, for example to track their medication intake, reduce insomnia or support physical activity.

Finally, further insights are required regarding the usefulness of different communication styles depending on the users’ specific goal and/or personal characteristics. While the present study revealed the differentiation between short-term and long-term goals as relevant, also the intensity of the desire to change or to improve oneself might affect the preferred or most effective style of communication. In sum, the present results already confirm the communication style of interactive products for self-improvement as an important aspect for a successful usage/change story. However, further research needs to substantiate the present findings and to provide grounding for the transformation of desired communication into design guidelines.

## Conclusion

Surprised by the lack of psychological foundation of many existing technologies for self-improvement (e.g., Conroy et al. 2014), the present research explored the dialog between product and user as a psychological factor with potentially high relevance. It showed that comparable to the dialog between coach and client, a product is only supportive in changing behavior if it speaks the “right language”. Different styles of communication come with particular emotional consequences, and thereby form one possible contributing factor for the success and endured use of self-improvement tools. We suggest models from communication psychology as a helpful frame to describe the user product dialog. The herein applied approach is not restricted to the field of tools for self-improvement and change. Instead, it provides a specific perspective on the dialog between product and user that may be helpful in different areas of positive computing or interactive technologies in general. As such, our work forms one example of how psychological theory can be utilized in the field of user experience research and technology for well-being. We hope that the present approach will be helpful and inspiring for others and can add to a more intense interdisciplinary exchange, sharing the common vision of seeing technology as a means to enhance people’s well-being.
